# Editorial: Estrogen receptors in endocrine disorders

**DOI:** 10.3389/fendo.2023.1325371

**Published:** 2023-11-01

**Authors:** Tiziana Filardi, Kristy L. Townsend, Rosamaria Lappano, Massimiliano Caprio

**Affiliations:** ^1^ Department of Human Sciences and Promotion of the Quality of Life, San Raffaele Roma Open University, Rome, Italy; ^2^ Department of Experimental Medicine, Sapienza University of Rome, Rome, Italy; ^3^ Department of Neurological Surgery, College of Medicine, The Ohio State University, Columbus, OH, United States; ^4^ Department of Pharmacy, Health and Nutritional Sciences, University of Calabria, Rende, Italy; ^5^ Laboratory of Cardiovascular Endocrinology, Istituto di Ricovero e Cura a Carattere Scientifico (IRCCS) San Raffaele, Rome, Italy

**Keywords:** estrogens, estrogen receptors, endocrine diseases, metabolic diseases, white adipose tissue, breast cancer, colorectal cancer, autoimmune thyroid diseases

Estrogens are sex hormones that play pivotal functions in both health and disease. Three different types of estrogen receptors (ERs) mediate estrogen actions: the nuclear estrogen receptor (ER)α and ERβ, and the membrane G protein-coupled estrogen-receptor-1 (GPER1). The broad distribution and multifaceted actions of ERs in mammalian tissues help explain the wide-ranging implications for estrogen signaling in numerous medical conditions, such as reproductive and bone disorders, cardiovascular disease, some cancers, and neurodegenerative disorders. In this scenario, the role of estrogens in women’s health is particularly evident after menopause, when their levels significantly decline, thus predisposing for metabolic disturbances like insulin resistance. This Research Topic includes two reviews and three original research manuscripts, which explored novel actions elicited by estrogens and their receptors in health and disease, giving emphasis to translational potential and gender differences.

The review by Chen et al. provides an overview of the structure and function of estrogens and ERs and their respective signaling pathways, highlighting the most relevant mechanisms behind their effects on multiple organs. The roles of ERs and their expression levels in different organs are discussed, emphasizing the disrupted mechanisms in the context of various diseases, disorders and conditions, ranging from cancer to cardiovascular disease. Of note, aside from the acknowledged ER dysregulation in cancer, ERs’ roles in other pathological conditions, including gastrointestinal and neurodegenerative diseases, are extensively covered in this review and highlight the need to further elucidate novel aspects of their physiological and pathophysiological roles.

In the second review of the Research Topic, Steiner and Berry. summarize current evidence about the multiple effects of estrogens on white adipose tissue (WAT), in an effort to better understand the disrupted mechanisms underlying metabolic diseases. The Authors have gleaned from the research literature that circulating estrogens contribute to body fat distribution and adipose remodeling, mainly via ERα and with sex-dependent hallmarks. They highlight that the bioavailability of estrogens is linked more with the metabolically healthy subcutaneous fat in the body. Steiner and Berry also discuss the inhibitory effects of estrogens on WAT hypertrophy, which is known to be associated with chronic low-grade inflammation, immune cell dysregulation, WAT fibrosis, and eventually insulin resistance. In the last section of the paper, the Authors examine the mechanisms implicated in the estrogen capability to promote WAT development, including the regulation of the adipocyte progenitor cell (APC) biology.

The interest in unravelling the roles of estrogen and ER signaling pathways in health and disease is piqued by the need to identify novel diagnostic and therapeutic targets. A deeper knowledge of the dysregulation of estrogen and ERs’ function in different pathological scenarios might significantly impact clinical decision-making. In the original research by Li et al., the prognostic utility of a 21-gene recurrence score (RS), which has been previously reported to predict recurrence of tamoxifen-treated, lymph node-negative breast cancer (1) was investigated for the first time in patients with ER positive (ER+) and human epidermal growth factor receptor 2 (HER2) negative (HER2-) breast cancer (BC) with positive lymph nodes. In particular, this genomic test was developed to evaluate the molecular profile of ER+/HER2- BC by analysing the expression of selected 21 genes, including those involved in estrogen signaling, in order to guide chemotherapy decision-making (Paik et al.). In a large cohort of patients, Li et al. observed that the RS testing was associated with better survival outcomes, independent of confounding variables, thus demonstrating a potential benefit of this tool in prognostic prediction and therapeutic decision in patients with ER+/HER2- BC with positive lymph nodes.

Recently, significant advancements in the knowledge of sex- and gender-differences across various medical conditions have been made. Importantly, a gender-based approach in the management of diverse diseases can pave the way for a tailored and more effective clinical care. In particular, epidemiological researches have suggested a gender influence on colorectal cancer (CRC) incidence, with lower figures in fertile women treated with hormonal replacement therapy, than in men. Expression levels of 17β-estradiol (E2) and progesterone (P) in CRC have been linked to better prognosis in preclinical studies. Nevertheless, there is scarce evidence about the potential utility of hormonal therapy in CRC. Mahbub et al. evaluated the anti-tumorigenic effects of E2 and P administration in CRC male mice. They also validated the findings of the animal studies in SW480 and SW620 human male colon cancer cell lines. The Authors provided evidence regarding the capacity of both E2 and P to enhance the ERβ and progesterone receptor (PR) signalling pathways, leading to suppressive effects on tumorigenesis. Furthermore, E2 and P monotherapy equally reduced tumor proliferation through the suppression of anti-apoptotic signals and the increased expression of pro-apoptotic proteins. Interestingly, the co-administration of the two hormones showed synergistic effects, therefore encouraging further studies to better explore the potential of hormonal therapy in the management of CRC in males.

Besides cancer, a sex dimorphism emerges in autoimmune diseases. There is robust evidence that women have heightened risk for autoimmune thyroid disease (AITD). Although the reasons behind gender differences in the pathogenesis of AITD are not yet known, it is acknowledged that estrogens and androgens differently influence the immune system, enhancing and suppressing immune response, respectively. In this regard, during development, the immune system is skewed between males and females, potentially due to both genetic and hormonal contributions (2). This has led to much speculation on whether prenatal exposure to sex hormones might be linked to the development of AITD later in life. On these bases, in the original research article by Święchowicz et al., the digit ratio (2D:4D), which is the relative lengths of the second (2D) and fourth (4D) digits and a biomarker of prenatal sex steroid exposure, was evaluated in female patients with Hashimoto thyroiditis (HT) and Graves’ disease (GD) versus female control subjects. In fetal life, high estrogen and low testosterone levels are thought to correlate with a high digit ratio. Patients with HT or GD displayed the highest and the lowest 2D:4D ratio, respectively, suggesting that a diverse hyperestrogenic or hyperandrogenic fetal *milieu*, might differently modulate immune system and thyroid function, resulting in an increased susceptibility to develop either HT or GD.

Overall, this Research Topic provides novel insights into the roles of estrogens and ERs in different conditions, with relevant translational implications and critical insights regarding biological sex differences. Aside from gynaecological cancer, estrogen/ER signaling is now known to play a pivotal role in the development of various diseases, emerging as a prospective therapeutic target in a wide range of conditions. Of note, the understanding of sex-differences in estrogen/ER modulation may pave the way for more gender-tailored research protocols and therapeutic strategies.

## Author contributions

TF: Writing – original draft. KT: Writing – review & editing. RL: Writing – review & editing. MC: Writing – original draft, Writing – review & editing.

## References

[1] Paik S Shak S Tang G Kim C Baker J Cronin M. A multigene assay to predict recurrence of tamoxifen-treated, node-negative breast cancer. N Engl J Med (2004) 351:2817–26. doi: 10.1056/NEJMoa041588 15591335

[2] Klein SL Flanagan KL. Sex differences in immune responses. Nat Rev Immunol (2016) 16(10):626–38. doi: 10.1038/nri.2016.90 27546235

